# Temozolomide-Induced RNA Interactome Uncovers Novel LncRNA Regulatory Loops in Glioblastoma

**DOI:** 10.3390/cancers12092583

**Published:** 2020-09-10

**Authors:** Sabrina Fritah, Arnaud Muller, Wei Jiang, Ramkrishna Mitra, Mohamad Sarmini, Monika Dieterle, Anna Golebiewska, Tao Ye, Eric Van Dyck, Christel Herold-Mende, Zhongming Zhao, Francisco Azuaje, Simone P. Niclou

**Affiliations:** 1 NORLUX Neuro-Oncology Laboratory, Department of Oncology, Luxembourg Institute of Health, Luxembourg L-1526, Luxembourg; sabrina.fritah@lih.lu (S.F.); mohamad.sarmini@lih.lu (M.S.); monika.dieterle@lih.lu (M.D.); anna.golebiewska@lih.lu (A.G.); francisco.azuaje@ucb.com (F.A.); 2 Quantitative Biology Unit, Luxembourg Institute of Health, Luxembourg L-1526, Luxembourg; arnaud.muller@lih.lu; 3 Department of Biomedical Informatics, Vanderbilt University Medical Center, Nashville, TN 37203, USA; bioccjw@yahoo.com (W.J.); santu.bioinf@gmail.com (R.M.); zhongming.zhao@uth.tmc.edu (Z.Z.); 4 Faculty of Science, Technology and Medicine, University of Luxembourg, L-4365 Esch-sur-Alzette, Luxembourg; 5 Institut de Génétique et de Biologie Moléculaire et Cellulaire (IGBMC), Centre National de la Recherche Scientifique, UMR7104, Institut National de la Santé et de la Recherche Médicale, U964, Université de Strasbourg, 67404 Illkirch, France; yetao@igbmc.fr; 6 DNA Repair and Chemoresistance, Department of Oncology, Luxembourg Institute of Health, Luxembourg L-1526, Luxembourg; eric.vandyck@lih.lu; 7 Experimental Neurosurgery, Department of Neurosurgery, University of Heidelberg, 69120 Heidelberg, Germany; h.mende@med.uni-heidelberg.de; 8 School of Biomedical Informatics, The University of Texas Health Science Center at Houston, Houston, TX 77030, USA; 9 Department of Biomedicine, University of Bergen, N-5009 Bergen, Norway

**Keywords:** glioblastoma, temozolomide, lncRNA, regulatory circuit, chemoresistance, transcriptome

## Abstract

**Simple Summary:**

Glioblastoma (GBM) is the most aggressive brain tumor and most resistant to therapy. The identification of novel predictive biomarkers or targets to counteract chemoresistance, requires a better understanding of the GBM primary response to therapy. The aim of our study was to assess the molecular response of GBM to the standard of care chemotherapy by temozolomide (TMZ). We established a comprehensive map of gene expression changes after treatment and discovered that GBM cells elicit a coordinated gene expression program after chemotherapy that differs between sensitive and resistant cells. We found that a novel class of genes expressed as long non-coding RNAs (lncRNAs) is involved in gene regulatory circuits in GBM and could represent novel markers of GBM patient prognosis. By shedding light on the involvement of the non-coding genome in GBM, our results may provide new mechanistic insight on lncRNAs and their importance in chemoresistance.

**Abstract:**

Resistance to chemotherapy by temozolomide (TMZ) is a major cause of glioblastoma (GBM) recurrence. So far, attempts to characterize factors that contribute to TMZ sensitivity have largely focused on protein-coding genes, and failed to provide effective therapeutic targets. Long noncoding RNAs (lncRNAs) are essential regulators of epigenetic-driven cell diversification, yet, their contribution to the transcriptional response to drugs is less understood. Here, we performed RNA-seq and small RNA-seq to provide a comprehensive map of transcriptome regulation upon TMZ in patient-derived GBM stem-like cells displaying different drug sensitivity. In a search for regulatory mechanisms, we integrated thousands of molecular associations stored in public databases to generate a background “RNA interactome”. Our systems-level analysis uncovered a coordinated program of TMZ response reflected by regulatory circuits that involve transcription factors, mRNAs, miRNAs, and lncRNAs. We discovered 22 lncRNAs involved in regulatory loops and/or with functional relevance in drug response and prognostic value in gliomas. Thus, the investigation of TMZ-induced gene networks highlights novel RNA-based predictors of chemosensitivity in GBM. The computational modeling used to identify regulatory circuits underlying drug response and prioritizing gene candidates for functional validation is applicable to other datasets.

## 1. Introduction

Since the introduction of temozolomide (TMZ) [1] in 2005 as standard chemotherapy against glioblastoma (GBM) [2], no additional drug has been identified to effectively slow down GBM progression. Unfortunately, however, median patient survival remains dismal and resistance to TMZ is inevitable. The epigenetic regulation of the O-6-methylguanine-DNA methyltransferase (MGMT) gene is the only known determinant of the clinical response to TMZ [3,4,5,6], eventually however, both MGMT methylated and unmethylated GBM develop resistance to TMZ. Additional resistance mechanisms are being investigated, including alternative DNA repair pathways, receptor tyrosine kinase or tumor suppressor (TP53, PTEN) signaling [7,8].

As studies of the transcriptional changes induced by TMZ mainly assessed long-term changes following the selection of resistant clones, the primary impact of TMZ on the GBM transcriptome remains elusive [9]. Moreover, available gene expression studies have traditionally been performed on classical adherent serum-dependent GBM cell lines [10,11], while it has been recognized that patient-derived tumor cells grown as three-dimensional spheres (commonly called glioma stem cells, GSCs) represent improved GBM models that display cellular and molecular gradients relevant for drug response studies [12,13]. 

Advances in next-generation sequencing (NGS) have revealed the importance of long non-coding RNAs (lncRNAs) as major components of the human transcriptome [14]. LncRNAs are pleiotropic regulators of transcription, acting as transcriptional enhancers [15] or scaffolds for chromatin remodeling complexes [16,17], while a subset of lncRNAs, known as competing endogenous RNAs (ceRNAs), can sequester miRNAs and prevent the binding of miRNAs to their mRNA targets [18,19]. LncRNAs are implicated in cancer [20,21], including GBM [22,23,24], yet few lncRNAs have been characterized at the functional level. Although a large number of lncRNAs are retrieved from sequencing data, the prioritization of lncRNAs for functional analysis remains a common challenge.

In this project, using sequencing of small and long RNAs, we established the transcriptomic changes induced by TMZ in patient-derived GBM stem-like cells [25], differing in drug sensitivity. We found that TMZ elicited different gene expression programs in sensitive versus resistant GBM cell lines and that the extent of transcriptomic alterations correlated with sensitivity to TMZ. In addition to mRNAs, TMZ induced a large number of regulatory RNAs, especially lncRNAs. We inferred and analyzed transcriptional regulatory circuits generated from the integration of small RNA-seq and RNA-seq datasets, which were further integrated with clinical and independent molecular data. We thus uncovered coordinated programs of drug response in GBM, i.e., regulatory circuits that involve transcription factors (TFs), mRNAs, miRNAs, and lncRNAs. Finally, we identified several lncRNAs that bear functional relevance for drug response whilst representing independent prognostic markers in GBM patients. 

## 2. Results

### 2.1. Differential DNA Damage Responses in GBM Stem-Like Cells Treated with TMZ 

**Figure 1:**
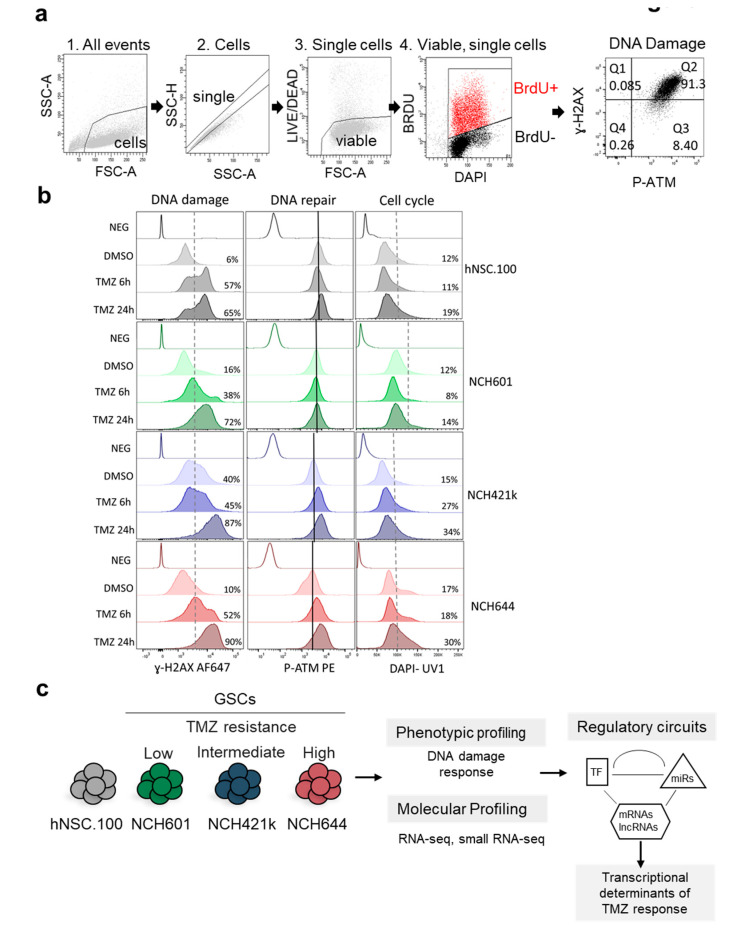
Glioblastoma (GBM) cells differentially activate DDR (DNA damage Response) in response to TMZ (temozolomide). (**a**) Gating strategy used for flow cytometry analysis. NCH644 GSCs (Glioblastoma stem-like cells) after 24 h TMZ treatment are shown as an example. (1) Cells were distinguished from debris using Forward Scatter (FSC) and Side Scatter (SSC). (2) Cell doublets and aggregates were gated out based on their properties displayed on the SSC area (SSC-A) versus height (SSC-H) dot plot. (3) Dead cells were recognized by their strong positivity for the dead cell discrimination marker. (4) Proliferating cells were distinguished as BRDU+ (Bromodeoxyuridine / 5-bromo-2’-deoxyuridine) events. DAPI (4′,6-diamidino-2-phenylindole)staining shows cell cycle profile. (5) DNA damage was assessed by measuring the levels of ɣ-H2AX whereas P-ATM (phosphorylated Ataxia telangiectasia mutated) was used to measure the extent of DDR activation. The gating applied discriminates between P-ATM positive vs. negative cells, and ɣ-H2AX high vs. low cells. Percentage of cells in each quartile is presented. (**b**) Response to TMZ was analyzed in GSCs (NCH601, NCH421k, and NCH644). Neural stem cells (hNSC.100) were used as a non-neoplastic control. DNA damage (ɣ-H2AX), DNA repair (P-ATM), and cell cycle profiles (DAPI) after TMZ treatment (TMZ 6 h and TMZ 24 h) or DMSO as vehicle, are measured in the indicated cell lines. Isotype controls for antibody staining are shown for each cell (Neg). Dotted lines separate low and high ɣ-H2AX cells, or G0/G1 and S/G2/M cell cycle (DAPI graphs). Percentage of ɣ-H2AX high cells and cells in the S/G2/M cell cycle phases is presented. Black lines indicate median expression of P-ATM in DMSO treated cells. (**c**) GBM stem-like cells of different chemoresistance to TMZ: Low (NCH601), Intermediate (NCH421k), and High (NCH644), and neural stem cells (hNSC.100) were further used for transcriptional profiling and systems level analysis.

We analyzed the response to TMZ of three patient-derived GBM stem-like cultures (NCH601, NCH421k, and NCH644, referred later as GSCs) [25,26] and of a human neural stem cell line (hNSC.100, referred later as NSCs) used as non-neoplastic control. We found that NCH644 GSCs, expressing the MGMT DNA repair enzyme, showed high resistance to TMZ (IC50 (half maximal inhibitory concentration) = 2.2 × 10^8^ nM vs. 2.7–3.7 × 10^5^ nM for the MGMT negative NCH421k and NCH601) (Figure S1a). Since TMZ is rapidly converted to the short-lived active compound MTIC, we hypothesized that a brief TMZ exposure could activate DDR. Therefore, we monitored established DDR markers using multi-color flow cytometry: phosphorylation of the histone variant H2AX on serine 139 (ɣ-H2AX) present at double strand DNA breaks and phosphorylation of Ataxia telangiectasia mutated (ATM) on serine 1981 (P-ATM) (Figure 1a). The basal levels of ɣ-H2AX were heterogeneous already prior TMZ treatment (Figure 1b, dotted line discriminates low and high subpopulations). NCH421k showed a high basal level (40% ɣ-H2AX high, Figure 1b), whereas NCH601 and NCH644 were similar to NSCs (6–16% ɣ-H2AX high). TMZ induced DNA double-strand breaks (ɣ-H2AX) after 6 h in three cell lines and after 24 h treatment in all cells (Figure 1b and Figure S1b). DDR activation as measured by P-ATM labeling was relatively homogeneous at baseline, with NCH644 showing the lowest basal level (Figure 1b, black lines indicate median expression in control conditions for each cell type). Upon TMZ treatment, NCH421k and NCH644 displayed P-ATM induction and accumulated in the S/G2/M phases of the cell cycle (Figure 1b), while NCH601 and NSCs showed little to no induction of P-ATM. In this experimental setting, we could not observe the presence of a sub-G1 peak after 24 h TMZ treatment indicative of apoptotic cells (Figure 1b). As expected, BrdU+ proliferative cells exhibited higher levels of ɣ-H2AX and P-ATM (Figure S1c). Taken together, our experiments revealed different cellular behavior upon TMZ (Figure 1c) from highly responsive (NSCs and NCH601: low basal level and strong induction of ɣ-H2AX, no induction of P-ATM), mildly responsive (NCH421k, high basal level and mild induction of ɣ-H2AX, low induction of P-ATM) to low responsive (NCH644, low basal level and strong induction of ɣ-H2AX, strong induction of P-ATM and S/G2/M accumulation). These results are in line with previous literature demonstrating the role of ATM in G2-M cell cycle block and protection from cell death, thereby leading to TMZ resistance [27]. These responses may reflect GBM cell heterogeneity and demonstrate the drug impact at an early time point. As we observed a differential DDR activation between the cell lines at 24 h after TMZ treatment, we therefore used these experimental conditions to further assess the primary transcriptional response to TMZ and identify the associated regulatory circuits (Figure 1c).

### 2.2. LncRNAs Are a Major Component of the Transcriptional Response to TMZ 

To determine the immediate transcriptional response of GBM to TMZ, we carried out RNA sequencing from cells treated for 24 h with TMZ or vehicle. From the same RNA samples, we performed small RNA sequencing, to allow the direct comparison of small RNA, mRNA and lncRNA expression levels, an essential experimental set-up for accurate data integration. 

**Figure 2:**
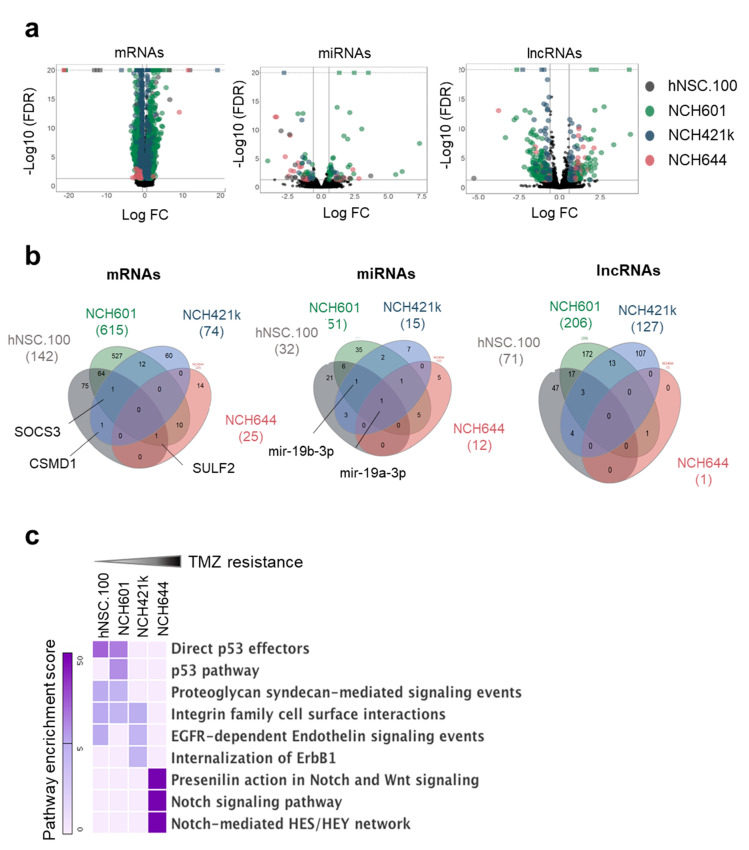
Genome-wide transcriptional changes induced by TMZ in GSCs. (**a**) Volcano plots representing the TMZ-regulated RNA subclasses in three GSCs (NCH601, NCH421k, NCH644) and one control neural stem cell line (hNSC.100): mRNAs (left panel), microRNAs (middle panel), lncRNAs (right panel). Each differentially expressed gene is represented by a dot and cell lines are indicated by different colors. Cut-offs for differential expression: FDR < 0.05 and absolute log FC > 2 for mRNAs, and FDR < 0.05 and absolute log FC > 1.5 for non-coding RNAs. Genes not satisfying these criteria are in black. For visualization purposes, the vertical axis has a limit of -log10 (FDR) = 20. (**b**) Venn diagrams showing the intersection of RNAs regulated by TMZ from the four cell lines. (**c**) Heat map representing the enrichment score of the top three pathways associated with mRNAs regulated by TMZ in each cell line.

Principal component analysis (PCA) identified individual variations between the respective GBM lines as the main determinant of transcriptome variability (Figure S2a). In both datasets, the variability measured by PC2 distinguished NCH644 cells from the other three cell lines and TMZ had a limited effect on variability. We compared the expression of different RNA biotypes with or without TMZ treatment (Table S1) and defined differential gene expression using a false discovery rate (FDR) < 5% and an absolute fold change > 2 for mRNAs and > 1.5 for miRNAs and lncRNAs. The volcano plots in Figure 2a represent the differentially expressed genes (DEGs) for mRNAs, lncRNAs, and miRNAs. In addition to the regulation of mRNAs, we observed that regulatory RNAs (miRNAs and lncRNAs) represented a large component of the transcriptome response elicited by TMZ (Figure 2a). 

The extent of the transcriptional response of GBM cells to TMZ was very different in NCH644 in comparison with the other GSCs and NSCs (Figure 2b). NCH601 cells, the most sensitive GBM in our selection, displayed the largest number of differentially expressed mRNAs (614), miRNAs (51), and lncRNAs (206), whereas in NCH644, the most TMZ resistant cell line, only 25 mRNAs, 12 miRNAs, and 1 lncRNA were significantly deregulated (Figure 2b and Table S2). By comparing the overlap of DEGs we found that individual gene regulation was not conserved across GSCs except for a limited number of genes e.g., *SOCS3*, *CSMD1*, and *SULF2* (Figure 2b). The same was true for miRNAs and lncRNAs. Interestingly, miR-19a-3p was the only common miRNA deregulated in all four cultures including NSCs, while miR-19b-3p regulation was observed in all cells except NCH644. 

We then asked if there was a coordinated TMZ-regulation of coding genes leading to statistical functional pathway enrichment [28]. Although there was limited overlap of differentially expressed mRNAs between cell lines, we identified several commonly enriched pathways in cells with similar response to TMZ (Figure 2c). For example, the p53 pathway was shared in sensitive cells (hNSC.100 and NCH601) whereas enrichment of Notch/Wnt signaling was present in the resistant NCH644. As all GSCs are TP53 WT (Figure S2b–d), the absence of p53 pathway induction in NCH421k and NCH644 upon TMZ is not due to a mutation in *p53*. In hNSC.100 we detected a heterozygous mutation p.K132M which is a described cancer missense mutation. The differential pathway enrichment in sensitive versus resistant GSCs is in line with the known role of *p53* in drug sensitivity and the involvement of developmental pathways (Notch/Wnt) in chemoresistance [29,30]. 

### 2.3. TMZ-Regulated LncRNAs Are Prognostic Markers of Overall and Disease-Free Survival for Glioma Patients

**Figure 3:**
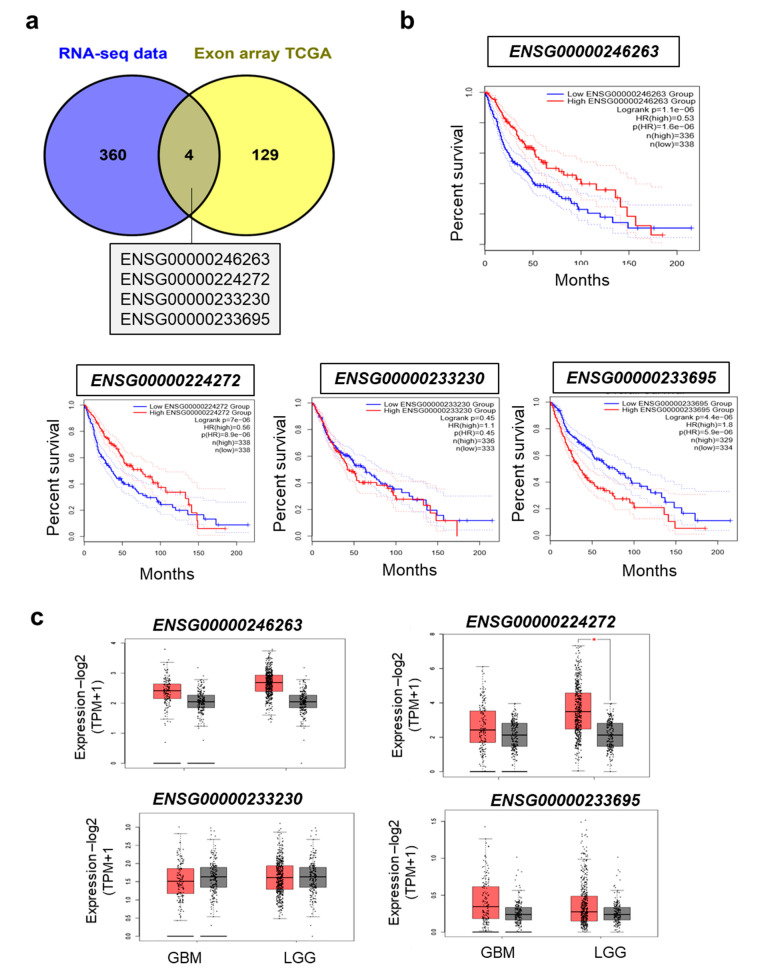
TMZ-regulated lncRNAs as novel independent GBM prognosis markers. (**a**) Overlap between the 360 TMZ-regulated lncRNAs and 133 lncRNAs proposed to correlate with GBM patient prognosis in the TCGA cohort [31]. (**b**) Kaplan Meier overall survival curves for the four overlapping lncRNAs in gliomas patients. The number of patients (*n*) analyzed for each group (high lncRNA expression group in red, low lncRNA expression group in blue) is indicated on the graphs. For each group *n* ≥ 329. Hazard ratios (H.R) and significance (log rank *p*-value) are indicated on the graphs. (**c**) Box plots of selected lncRNA median expression in GBM (*n* = 163) and lower grade gliomas (LGG, *n* = 518) (in red) compared to control brain tissue (grey, *n* = 207). Significant expression change is indicated by a red asterisk.

A previous analysis of lncRNA expression on exon arrays from The Cancer Genome Atlas cohort (TCGA) revealed 133 lncRNAs as putative prognostic markers in GBM [31]. Four of our 364TMZ regulated lncRNAs were also found in this dataset: *ENSG00000224272*, *ENSG00000233230*, *ENSG00000233695* (or *GAS6-AS1*), and *ENSG00000246263* (Figure 3a). Using the webtool GEPIA [32], we calculated their prognostic capacity on glioma overall (Figure 3b) and disease-free survival (Figure S3a), and confirmed the positive prognostic value for *ENSG00000246263* and *ENSG00000224272* but not for *ENSG00000233230*. On the other hand, a high expression of *GAS6-AS1* was a negative prognostic factor in gliomas, as previously observed in gastric cancer [33]. We next compared the expression of these four lncRNAs in GBM, lower grade gliomas (LGG), and normal brain (GTEX). The median expression of each lncRNA was not significantly different between LGG and GBM or between brain tumors and normal brain, except for *ENSG00000224272,* which was more expressed in LGG than normal brain (Figure 3c). We then searched if the lncRNA expression differed in GBM subgroups of better prognosis, taking into account either the promoter methylation of MGMT (GBM patients who benefit from TMZ) or GBMs harboring a DNA hypermethylation gene signature (CIMP phenotype). Within the GBM cohort, we found that the expression of the four selected lncRNAs was independent of MGMT status (Figure S3b) and the CIMP phenotype (Figure S3c). In summary, we identified novel lncRNAs that are regulated by TMZ and display prognostic value in GBM, suggesting a role in tumor progression and response to therapy.

### 2.4. Computational Inference of TMZ-Associated RNA Interactome 

A common challenge in noncoding RNA research is to predict the biological significance of newly identified molecules and select key genes for functional validation. As noncoding RNAs exert major functions in transcriptional regulation, we sought to uncover regulatory networks underlying the GBM response to TMZ. To this aim, we developed a pipeline based on available information on lncRNA and mRNA associations with transcription factors (TFs) and miRNAs. 

**Figure 4:**
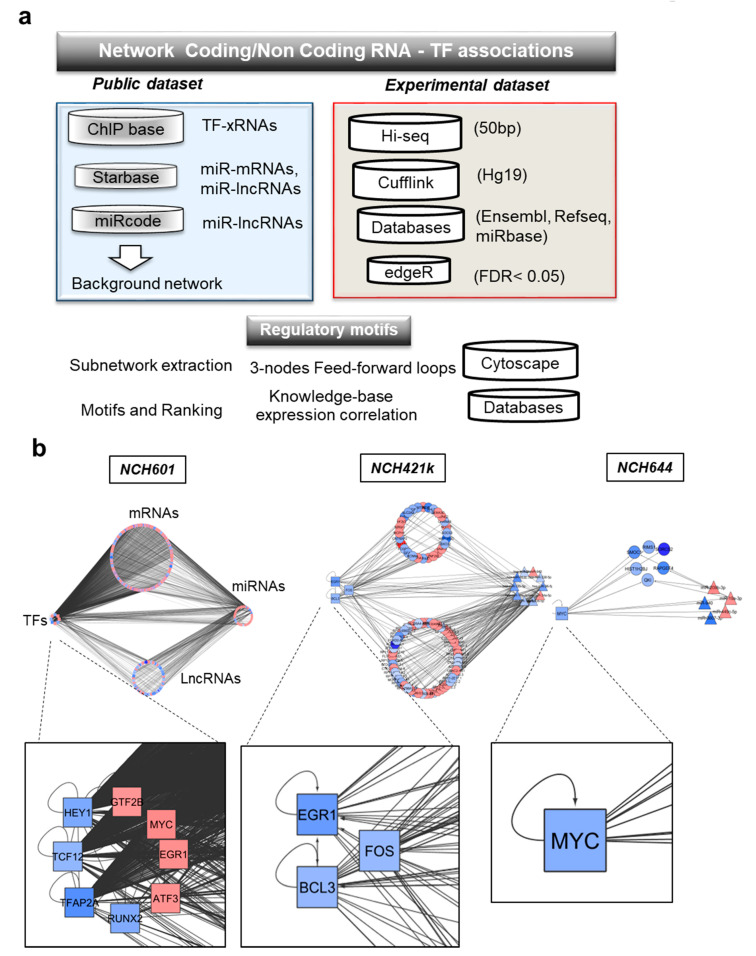
Systems approaches used for the generation of the RNA interactome and gene regulatory networks induced by TMZ in GBM. (**a**) Overview of the analysis pipeline, (**b**) gene regulatory networks representing the molecular associations between different RNA biotypes in different GSCs: mRNAs, miRNAs, lncRNAs, and transcription factors (TFs). Up- and downregulated RNAs are shown in red and blue, respectively. Different node shapes distinguish RNA biotypes: TF: square, miRNA: triangle, mRNA: circle, lncRNA: hexagon.

To build such a global gene regulatory network of mRNAs, ncRNAs (miRNAs and lncRNAs), and TFs, we first retrieved molecular association data from several databases: ChipBase, StarBase, and miRcode databases (Figure 4a) [34,35,36]. TF-mRNA and TF-lncRNA associations from ChipBase were based on Chip-seq data of TF binding to promoters of mRNAs and lncRNAs. From Starbase, we extracted miRNA-mRNA/lncRNA associations from HITS-CLIP and PAR-CLIP experiments. Additional computationally predicted miRNA-lncRNA associations were added from miRcode. After gene annotation using Refseq for mRNAs, miRBase release 20 for miRNAs, and Ensembl for lncRNAs, the molecular associations described in Figure 4a were combined to obtain a global background network that we named the “RNA interactome”. This exhaustive network contains regulatory interactions among 107 TFs, 1851 miRNAs, 10’970 lncRNAs, and 18′812 mRNAs (Table S3).

Next, we mapped our experimental dataset of TMZ-induced DEGs on the RNA interactome and extracted specific networks. In these networks, mRNAs coding for transcription factors were labeled as TFs. These analyses resulted in cell line-specific subnetworks composed of RNA-RNA and/or TF-RNA interactions. Cell line subnetworks were visualized as circular layouts (Figure 4b) using cytoscape, where each cluster represents the RNA biotype (TFs, mRNAs, lncRNAs, or miRNAs). Inside each cluster, nodes represent individual genes, with the sense of TMZ-induced deregulation indicated by a color code (increased expression in red; decreased expression in blue) (Figure 4b). We found that some TMZ-regulated molecules appeared to organize in network hubs of highly connected genes (Table S4). Furthermore, transcriptional responses to TMZ involved a great number of TF-mRNAs and TF-lncRNAs interactions. These interactions involved the following transcription factors: MYC, TFAP2A, TCF12, HEY1 and EGR1, FOS and BCL3 (Table S4). Gene regulatory networks were largely composed by ncRNA interactions (miRNAs-mRNAs or miRNAs-lncRNAs) regulated by a specific subset of miRNAs. Several DEGs, as for example MYC and miR-19a-3p, showed opposite regulation by TMZ in the sensitive NCH601 and resistant NCH644 cell lines. This may indicate that the specific gene regulatory network contains key regulators of TMZ response.

We next analyzed the connectivity between the DEGs. We identified complex subnetworks in NCH601 and NCH421k that displayed a large number of DEGs. In contrast, only a small subnetwork was detected in NCH644, which displayed relatively few TMZ-associated DEGs, with some of its components being also present in NCH601 networks. We then measured the connectivity of each node (i.e., a gene) by calculating the in-degree (number of interactions that target a node) and out-degree of each node (number of out-going edges from a node). Notably, miR-19a-3p and miR-19b-3p displayed high out-degree values in almost all cells, suggesting a prominent regulatory role for these miRNAs in TMZ treatment response. 

In parallel, we searched for putative promoter activities that may explain the observed transcriptional changes using ISMARA web tool [37]. The analysis of promoter motifs highlights TFs with different activity in response to TMZ without changes in their expression levels. We analyzed the top 10 transcription factor motifs sorted by activities (*z*-value) and identified common promoter motifs as for example YBX1, NRF1, and SP1 (Figure S4). Whereas this observation indicates that common transcriptional regulators can react in response to TMZ, it also suggests that they do so in opposite direction in resistant and sensitive cells: if they are activated/unchanged in responsive cells (NSC and NCH601), they tend to be repressed in resistant cells (NCH421k and NCH644). In summary, our genome-wide view of the transcriptional response to TMZ discerned sensitive and resistant behaviors. 

Taken together, these data show that TMZ treatment impacts not only mRNA expression, but also miRNA and lncRNA levels, which may constitute regulatory networks with other RNA biotypes and TFs. Such regulatory networks, which were mostly observed in sensitive cell lines, may provide novel insight into chemosensitivity of GBM cells.

### 2.5. TMZ-Induced Transcriptional Regulatory Motifs Contain LncRNAs 

**Figure 5:**
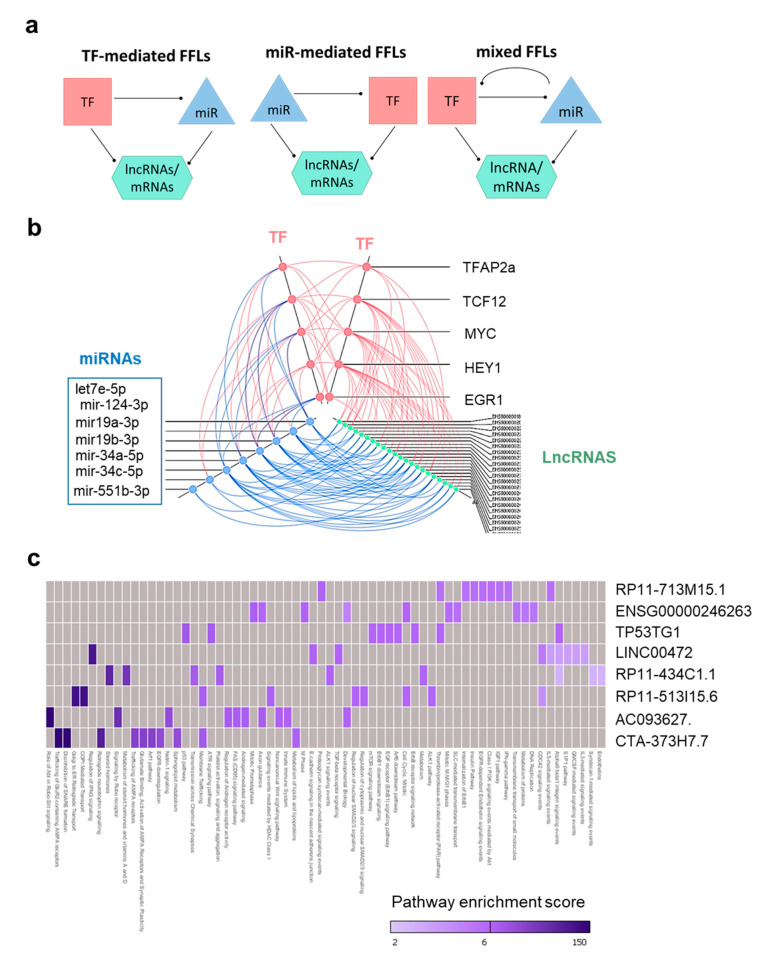
Identification of TMZ-associated transcriptional regulatory motifs involving lncRNAs. (**a**) Graphical representation of the motif structures where mRNAs or lncRNAs are the targets regulated by miRNA (miR) and TF associations. (**b**) Hive plot representing TMZ-regulated FFLs containing lncRNAs from NCH601. Axes indicate different RNA families, with each dot corresponding to a gene (TF, miRNA, or lncRNA) involved in lncRNA-containing loops. Molecular interactions are represented by a color code line (stimulatory interactions in red, inhibitory interactions in blue). (**c**) Heatmap showing enriched pathway for the indicated lncRNAs.

To identify critical components of the transcriptional regulation elicited by TMZ, we analyzed cell line specific subnetworks at a higher systems level and extracted feed-forward loops (FFLs) involving TFs, miRNAs, mRNAs, or lncRNAs (Figure 5a). Such loops represent three-component interaction motifs consisting of miRNA-mediated loops, TF-mediated loops or mixed loops, i.e., those involving a dual interaction between TFs and miRNAs (Figure 5a). Transcriptional motifs were only present in the GBM cell lines, as no TF was regulated in NSC upon TMZ. Although the NCH644 network included a limited number of TMZ-regulated transcripts, we found two TF-mediated motifs with mRNAs involving MYC and miR-19a-3p. Conversely, in the TMZ sensitive cell line NCH601, we identified 308 miRNA-mediated loops, 559 TF-mediated loops, and 136 mixed loops regulated by TMZ (see Table S5 and Figure S5a). Strikingly, miR-19a-3p associated motifs were present in all GBM cell lines, although these associations involved different TFs, mRNAs, or lncRNAs depending on the cell line (Table S5).

We focused on the composition of motifs involving lncRNAs, as such motifs were only present in the most sensitive GSCs (NCH601). The 22 lncRNAs formed 104 loops by interaction with a limited number of regulators (five TFs and six miRNAs, Figure 4b), as visualized by the hive plot in Figure 5b. Notably, a small number of key regulators such as miR-19a/b, MYC, EGR1, and HEY1 were consistently present in most of the lncRNA-containing loops. To verify the non-coding identity of these 22 lncRNAs, we retrieved their sequences [38], calculated their coding potential probabilities [39], and compared them with those obtained from reference sets of known lncRNAs and mRNAs [40]. The lncRNAs involved in TMZ-associated regulatory loops had a low coding probability, similar to other known lncRNAs, suggesting that these lncRNAs are indeed noncoding (Figure S5b). 

Among the 22 lncRNAs involved in loops, eight were expressed in all RNA-seq samples. By calculating the expression correlation coefficient between lncRNAs and mRNAs (Table S6) and pathway enrichment analysis using Webgestalt, we were able to associate these eight lncRNAs with putative biological functions. Analysis of the top 10 pathways identified for each lncRNA based on their statistical significance revealed that several pathways were shared (Figure 5c). Thus, five lncRNAs were associated with cell cycle regulation and DDR and six lncRNAs with EGFR signaling and/or developmental function (Figure 5c). As these functions were also enriched at the mRNA level (Figure 2c), our data collectively strengthen the notion that the motif-specific lncRNAs identified by our analysis are involved in biological functions relevant to drug response. 

### 2.6. LncRNAs in TMZ Regulatory Loops May Function as CeRNAs

**Figure 6:**
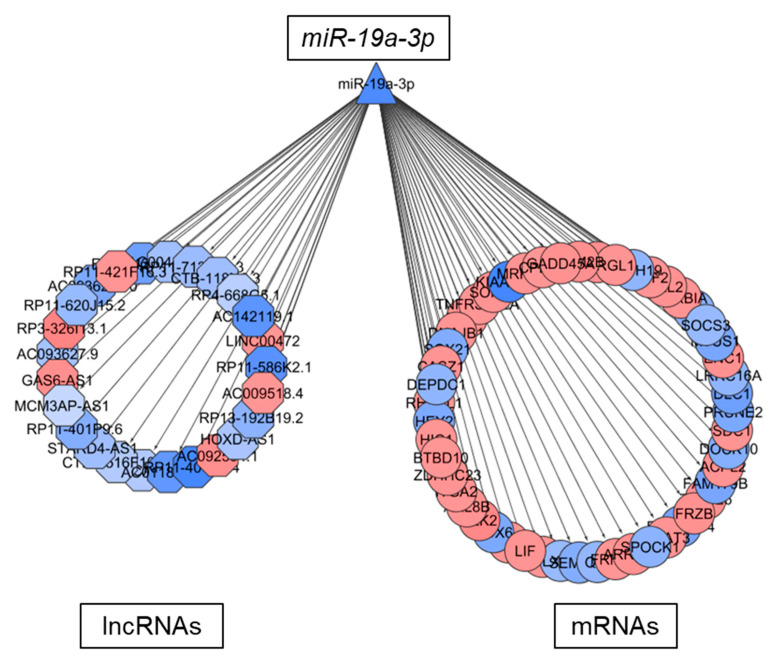
Putative ceRNA function of lncRNAs regulated by TMZ. Gene regulatory network representing the molecular associations between miR-19a-3p and mRNAs or lncRNAs in NCH601. Up- and downregulated RNAs are shown in red and blue, respectively. Different node shapes distinguish RNA families; TF: square, miRNA: triangle, mRNA: circle, lncRNA: hexagon.

A subset of lncRNAs signal through binding of miRNAs, thereby acting as competing endogenous RNAs (ceRNAs) with mRNAs. We investigated the TMZ associated gene regulatory networks and selected mRNA-miRNA and miRNA-lncRNA pairs for the seven miRNAs involved in TMZ regulatory loops (Figure 5b): let7e-5p, miR-124-3p, miR-19a-3p, miR-19b-3p, miR-34a-5p, miR34c-5p, and miR-551b-3p. Interestingly, we found that except for miR-551b-3p (Figure S6), all miRNAs investigated shared molecular interactions with mRNAs and lncRNAs (Figure 6 and Figure S6). Taken together, these results suggest that these lncRNAs may act as sponges for miRNAs to fine tune the expression of mRNAs regulated by TMZ. 

## 3. Discussion

The transcriptional response to chemotherapeutic drugs has so far been mostly analyzed at the level of mRNAs, yet, a wealth of information is present in non-coding RNAs, making up more than 90% of the human genome [41]. Here, we report that GSCs elicit a heterogeneous cellular and transcriptional response to acute TMZ exposure. Although TMZ induced DNA damage in all cells, DDR activation was only observed in the TMZ-resistant MGMT positive GSC. Yet, this strong DDR activation was not associated with a major deregulation of the transcriptome in the resistant GSC, which is in line with our recent study where TMZ did not induce phenotypic changes in this cell line [42]. At present we cannot evaluate if and to what extent this is linked to MGMT expression in this cell line. In our experimental conditions, we showed that TMZ induces cell cycle block in some GSCs and we did not detect TMZ induction of apoptosis (subG1 peak) in GSCs, which is in line with previous report [43,44]. As other studies have evidenced apoptosis induction by TMZ in GBM cells, or more recently ferroptosis [6,45,46,47], it would be interesting to assess if the transcriptional changes and regulatory circuits identified here are involved in TMZ mediated apoptosis.

Our study provides a comprehensive overview of the RNA regulatory circuits induced by TMZ and lays the basis for exploring non-coding RNA function in GBM tumorigenesis and chemosensitivity. The analysis of regulatory network identified key TF-miRs molecular interactions, as the crosstalk between mir-19 and MYC [48]. Mir-19, a key oncogenic miRNA involved in glioma proliferation, invasion, and progression [49,50,51], appears to be a central regulator of the transcriptional response to TMZ. The upregulation of mir-19 in the resistant GSCs could participate in chemoresistance. Of note, it was reported that mir-19 targeting decreased the expression of MYC and delayed GBM tumor growth [52]. The systems-level characterization of lncRNAs and their inclusion in human-curated databases are still at an early stage. A study investigating a ceRNA network, which was built with co-expression measurements from paired genes (mRNAs and lncRNAs), proposed lncRNAs with potential clinical relevance in GBM [53]. The present approach expands on a network generation and analysis strategy that we previously reported in the context of miRNAs and GBM [54]. This strategy resulted in a comprehensive integration of datasets and regulatory associations, which is required to provide systems-level insights into specific cell models and treatment responses. An important challenge for such a strategy is the lack of tools for full integration of the different modeling and analysis steps. Another obstacle is the limitation imposed by the relatively poor consistency of ncRNA annotations between databases [55]. The reannotation of existing patient datasets with lncRNA expression [31] and sequencing of total RNAs could enhance the phenotypic studies of lncRNAs and the predictive potential of network-based discovery strategies. Nevertheless, the strategy presented here can be applied to multiple fields of cancer research investigating transcriptional programs (treatment resistance, cell invasion, immuno-oncology), whilst also contributing to the development of appropriate methodologies and analytical tools. In this regard, it is notable that the methodology we implemented to prioritize lncRNA candidates for functional validation resulted in the identification of a subset of lncRNAs with a putative role in DDR or developmental pathways. Among these novel lncRNAs, the lncRNAs TP53TG1 and ENSG000246263, were recently uncovered in a study using a machine-learning method to stratify cancer-related lncRNAs [56]. Furthermore, the documented roles of TP53TG1 in glioma cell proliferation [57] and response to chemotherapy in lung cancer [58] provide experimental support for our in silico prediction of a role for TP53TG1 in the response of GBM cells to TMZ.

Our work has revealed that a large number of lncRNAs are differentially regulated by TMZ in chemo-sensitive and resistant GSCs, as well as in non-malignant NSCs, and identified key lncRNAs that may be linked to the regulation of DDR, apoptosis, and EGFR signaling in GBM, as part of co-expression networks with mRNAs and other small RNAs. Although several DNA repair mechanisms are involved in the repair of TMZ-induced lesions, TMZ resistance in the clinic has so far been associated mainly with the activity of MGMT and the selection of resistant clones that account for tumor recurrence [8]. To date, albeit recent reports involve lncRNAs as regulators of drug sensitivity [59,60,61] and of GBM pathogenesis [22,23], a genome-wide analysis of lncRNAs in the regulation of drug response in GBM has not been done. We expect that functional analyses of TMZ-associated lncRNAs will provide valuable insight into the mechanisms that govern drug response in GBM. Given the importance of lncRNAs in cellular lineage and as transcriptional enhancers, it is highly possible that some of the lncRNAs identified in this study operate through epigenetic mechanisms that drive selection of resistant cells, independently of MGMT. In this respect, we identify four lncRNAs with prognostic value in glioma patients that are involved in the transcriptional response to TMZ, independent of MGMT status. Although lncRNAs are generally expressed at low levels, it would be of interest to verify their prognostic value by RNA in situ hybridization on glioma tissue. Additionally, as lncRNAs play important roles in DNA damage signaling and chemoresistance, these molecules could have a functional role in TMZ response independently of MGMT as recently shown for the lncRNA TP73-AS1 [62]. If confirmed, targeting these molecules could be used to counteract chemoresistance in GBM. 

## 4. Material and Methods

### 4.1. Cell Lines, Treatments, and Cytotoxicity Assay

The glioblastoma stem-like cell cultures NCH421k, NCH601 and NCH644 were generated in the lab of Dr Christel Herold-Mende (Department of Neurosurgery, University of Heidelberg) [63] and characterized previously [25]. Human fetal neural stem cells hNSC.100 were described previously [64].All cell lines were grown as 3D neurospheres in serum-free medium. NCH421k and NCH601 were cultured as non-adherent spheres in DMEM-F12 medium (Lonza, Basel. Switzerland) containing 1xBIT100 (Provitro, Berlin, Germany), 2mM L-Glutamine, 30U/ml Pen-Step, 1U/ml Heparin (Sigma), 20ng/ml bFGF (Miltenyi, Bergisch Gladbach, Germany 130-093-841) and 20ng/ml EGF (Provitro, Berlin, Germany 1325950500). NCH644 and hNSC.100 grew as non-adherent spheres in Neurobasal® base medium (ThermoFischer Scientific, Waltham, Massachusetts, USA) supplemented with 1xB-27, 2mM L-Glutamine, 30U/ml Pen-Step, 1U/ml Heparin (Sigma), 20ng/ml bFGF and 20ng/ml EGF. Temozolomide (TMZ, Sigma Aldrich, Overijse, Belgium, 76899) sensitivity was measured by WST-1 assay (Roche, Basel, Switzerland, 5015944001) developed with Fluorostar Optima system. IC50 was determined using Graphpad Prism. Cell biomass was determined 72 h after TMZ treatment. Cells were treated with 500 µM TMZ for 6 or 24 h for flow cytometry, and 24 h for RNA-seq experiments.

### 4.2. Multi-Color Flow Cytometry 

TMZ or vehicle were added 24 or 6 h prior to analysis. For proliferation assay, BRDU (Bromodeoxyuridine / 5-bromo-2’-deoxyuridine) incorporation was performed for 6 h before the end of the experiment. Prior to fixation, cells were dissociated and incubated with the IR-LIVE/DEAD® Fixable Dead Cell Stain (ThermoFisher scientific; Waltham, MA, USA, 1 µg/mL). Cells were fixed, permeabilized and stained with the BD Pharmingen^TM^ BrdU Flow Kit (BD Bioscience, San Jose, CA, USA) according to the manufacturer’s instructions. The following conjugated antibodies were used: anti H2AX-Phospho Ser139-Alexa Fluor 647 (BD Biosciences, 560447), anti-ATM-PhosphoSer1981-PE (Millipore, Burlington, MA, USA, FCMAB110P), Alexa Fluor® 647 Mouse IgG1 κ Isotype Control (BD Bioscience, 565571), Mouse IgG1 Negative Control, clone Ci4, PE conjugate (Millipore, MABC002H).DNA was counterstained with DAPI (1 ug/mL) and anti–BrDU-PerCP-Cy5.5 (BD, 560809 kit). Data were acquired on a FACS Aria^TM^ SORP cytometer (BD Biosciences) and analyzed with Diva (BD Biosciences) and FlowJo software version v10.5.3. 

### 4.3. RNA and Small RNA-Sequencing

Total RNA was extracted with Trizol and quantified using Nanodrop. Three biological replicates were used per experimental condition. RNA quality was checked using a bioanalyzer (Agilent, Santa Clara, CA, USA). Total RNAs were depleted from Ribosomal RNAs using RiboMinus™ technology. RNA-seq libraries were prepared according to the Illumina standard protocol using TruSeq Stranded RNA Kits. Small RNA-Seq libraries were generated from total RNA using TruSeq Small RNA Library Prep Kit. Single stranded sequencing reads were performed on HiSeq2500 instrument (Illumina, San Diego, CA, United States). Base calling was performed with CASAVA 1.8.2 pipeline (Illumina). Fastq data and processed counts of RNA-seq (24 samples in total) and small RNA-seq (24 samples in total) are available through Gene Expression Omnibus (GSE98128). 

### 4.4. Systems Approaches

The associations of TFs with genes, miRNAs, or lncRNAs were obtained from ChipBase [36]. Associations between miRNAs and genes were obtained from StarBase [35]. As the number miRNAs-lncRNAs associations in StarBase was small, we also integrated the miRcode dabase [34]. As a result, the background regulatory network consisted of 1,145,815 regulations, including 107 TFs, 1851 mature miRNAs, 10,970 lncRNAs, and 18,812 genes. Detailed information is shown in Figure 4B. Next, we mapped the DEGs (Differentially Expressed) genes, miRNAs, and lncRNAs of each cell line into the background network separately. We constructed the cell line-specific subnetworks by extracting the edges (observed expression correlation) between the DE nodes. We focused on three types of 3-node FFLs containing lncRNAs or mRNAs, and which included a TF, a miRNA, and a target lncRNAs or mRNAs (Figure 5a and Figure S5, respectively). In the first type, a TF regulates miRNA and lncRNA, and a miRNA regulates lncRNA. We termed it TF-mediated FFL. In the second type, miRNA-mediated FFL, a miRNA regulates TF and lncRNA, and a TF regulates lncRNA. In the third type, a miRNA and a TF are mutually regulated, and both regulate a lncRNA. We extracted all FFLs from the cell line-specific subnetworks using R-language scripts.

### 4.5. TCGA Data Analysis

Prediction of lncRNAs as potential prognostic markers were reported in [31], analyzed using GEPIA, and GETX data were used for normal tissue expression. The MGMT promoter methylation status was from [65]. Cohort was divided in two groups based on expression median for each lncRNA. Group expression was represented using Graph Pad Prism or extracted from GEPIA. 

## 5. Conclusions

In conclusion, this study provided a comprehensive map of transcriptome regulation upon TMZ in GSCs displaying different drug sensitivity. The transcriptional response to TMZ is a coordinated program of coding and non-coding RNAs, orchestrated by regulatory loops, some of them being oppositely modulated in sensitive and resistant GBM cells. Importantly, we uncovered a subset of largely unknown lncRNAs potentially involved in essential pathways of tumorigenesis and drug resistance. Several TMZ regulated lncRNAs display prognostic value in GBM patients. This data resource, the systems approaches, and novel RNA targets identified in this study open the way for understanding lncRNA function in GBM.

## Supplementary Materials

The following are available online at https://www.mdpi.com/2072-6694/12/9/2583/s1, Figure S1 TMZ sensitivity and DDR activation in GSCs, Figure S2: Principal Component analysis of all samples and TP53 genomic status of GSCs, Figure S3: TMZ-regulated lncRNAs as novel independent GBM prognosis markers of progression free survival, Figure S4: Selected examples of Top 10 transcription factor motifs sorted by activities (z-value) from ISMARA Figure S5: mRNA-containing regulatory loops and coding potential of selected lncRNAs, Figure S6: Gene regulatory networks representing the molecular associations between selected miRNAs and mRNAs or lncRNAs in NCH601, Table S1: All expression data, Table S2: Differentially expressed genes per biotype and cell line, Table S3: Regulatory background network, Table S4: Degree in subnetworks, Table S5: Transcriptional motifs summary, Table S6: Expression correlation calculation.
