# Supplementary material for: Temozolomide-Induced RNA Interactome Uncovers Novel LncRNA Regulatory Loops in Glioblastoma
Source: Cancers (Basel). 2020 Sep 10;12(9):2583. doi: 10.3390/cancers12092583 (PMC7563839; doi:10.3390/cancers12092583)
Supplement: Supplementary file 1 [file cancers-12-02583-s001.zip › cancers-887040 suppl final.docx]

**Supplementary Materials:**

Temozolomide-Induced RNA Interactome Uncovers Novel LncRNA Regulatory Loops in Glioblastoma


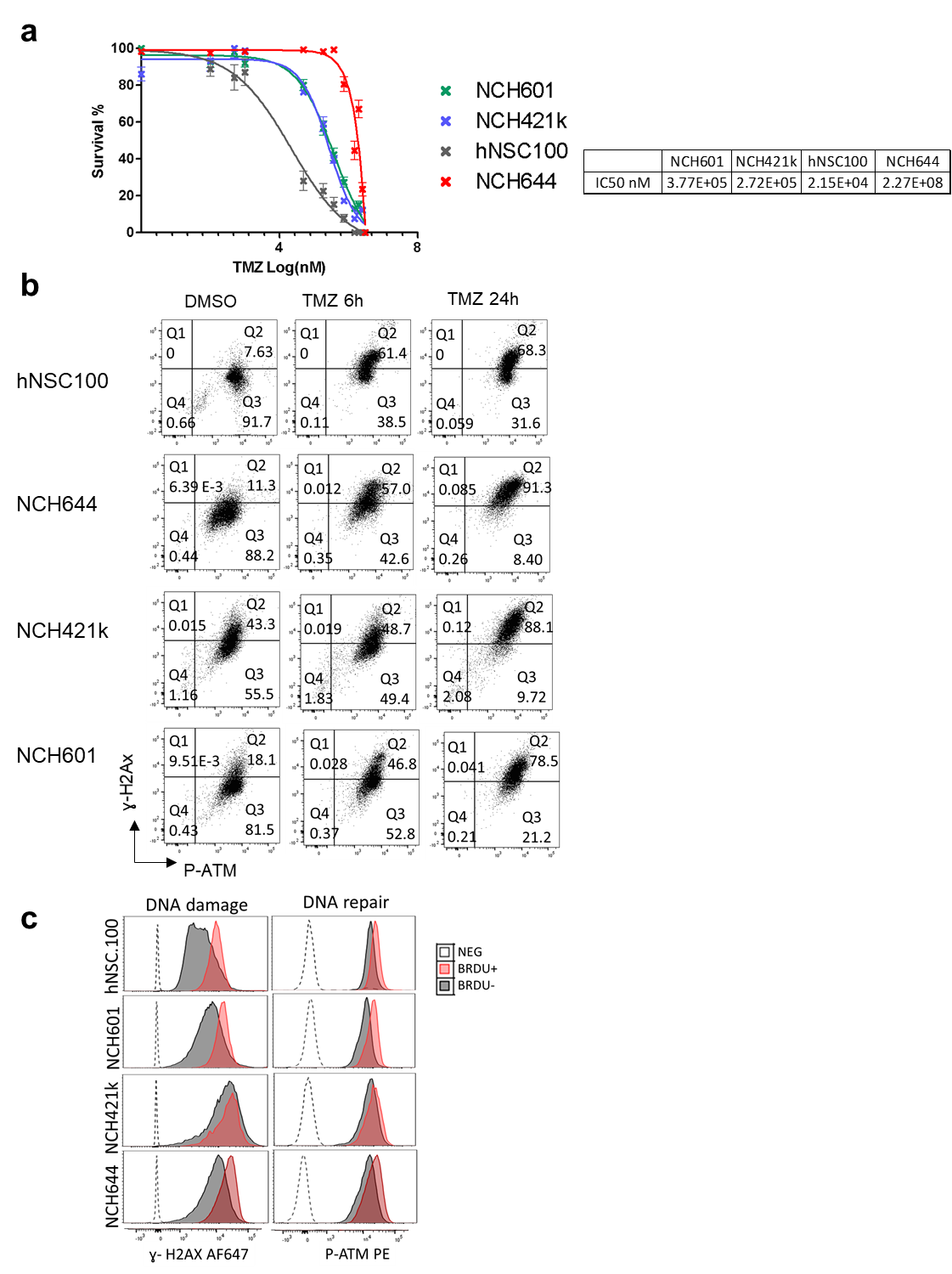


**Figure S1**. TMZ sensitivity and DDR activation in GSCs. (**a**) IC50 after 72 h of TMZ treatment (**b**) Dot plots showing basal level and induction of DNA damage (ɣ-H2AX) and DNA repair (P-ATM) after 6 hours and 24 hours TMZ treatment, or 24 h DMSO. The gating applied discriminates between P-ATM positive vs. negative cells, and ɣ-H2AX high vs low cells. Percentage of cells in the four quartiles are shown. (**c**) Basal level of ɣ-H2AX and P-ATM is higher in BRDU+ cells than BRDU- cells. Isotype controls for antibody staining are shown for each cells (Neg).


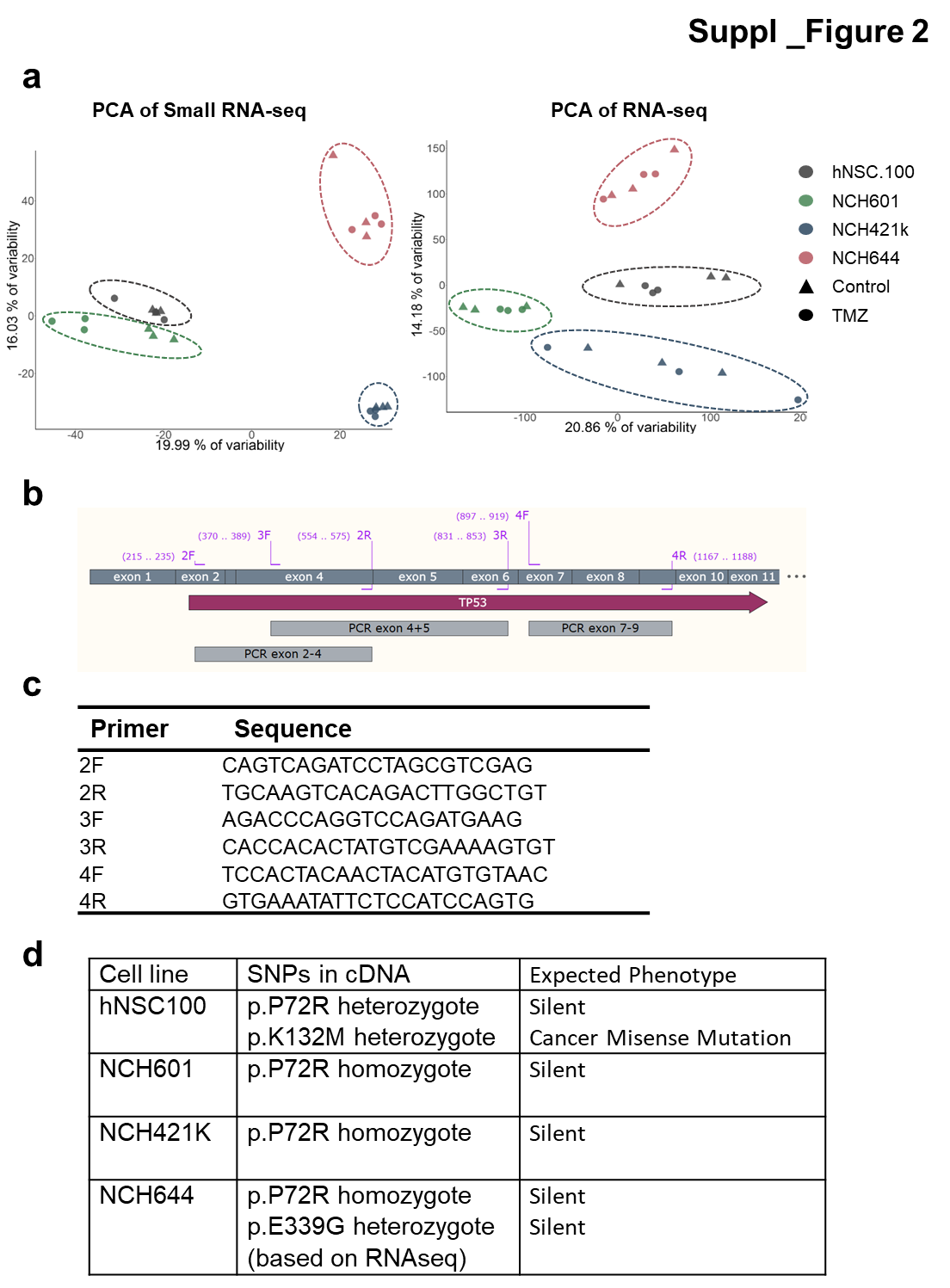


**Figure S2.** Principal Component analysis of all samples and TP53 genomic status of GSCs (**a**) Principal component analysis of Small RNA-seq and RNA-seq data: the cell line is the main source of transcriptome variation. Three biological replicate have been analysed per condition. Cell lines are shown as followed: hNSC100 are in grey, NCH601 in green, NCH421k in blue, NCH644 in pink; control samples are in triangle and treated samples as circles. (**b**) The scheme indicates the position of the primers used and the sequenced PCR fragments, (**c**) Table with primer sequences used for PCR, (**d**) Table with detected SNPs and related phenotype based on IARC database.


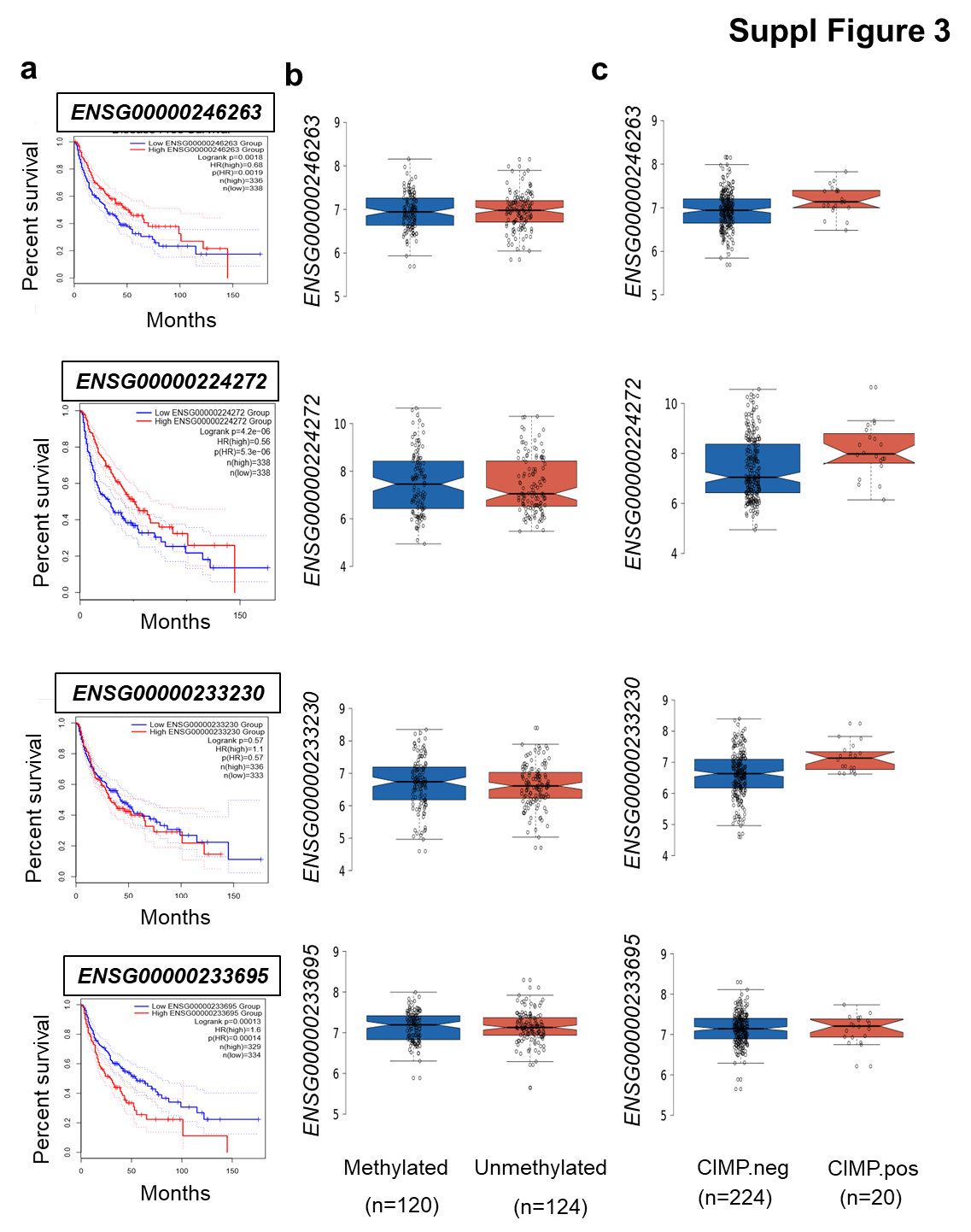


**Figure S3.** TMZ-regulated lncRNAs as novel independent GBM prognosis markers of progression free survival (**a**) Kaplan Meier disease-free survival curves for the 4 overlapping lncRNAs in gliomas patients. Significance is indicated by log rank p-value on the graphs (**b**) and (**c**) Box plots of lncRNAs in GBM patients with methylated (*n* = 120) or unmethylated MGMT (*n* = 124) in (b), as well as GBM patients with positive (*n* = 20) or negative CIMP phenotypes (*n* = 224) in (c) .Significance is indicated by non-overlapping notches.


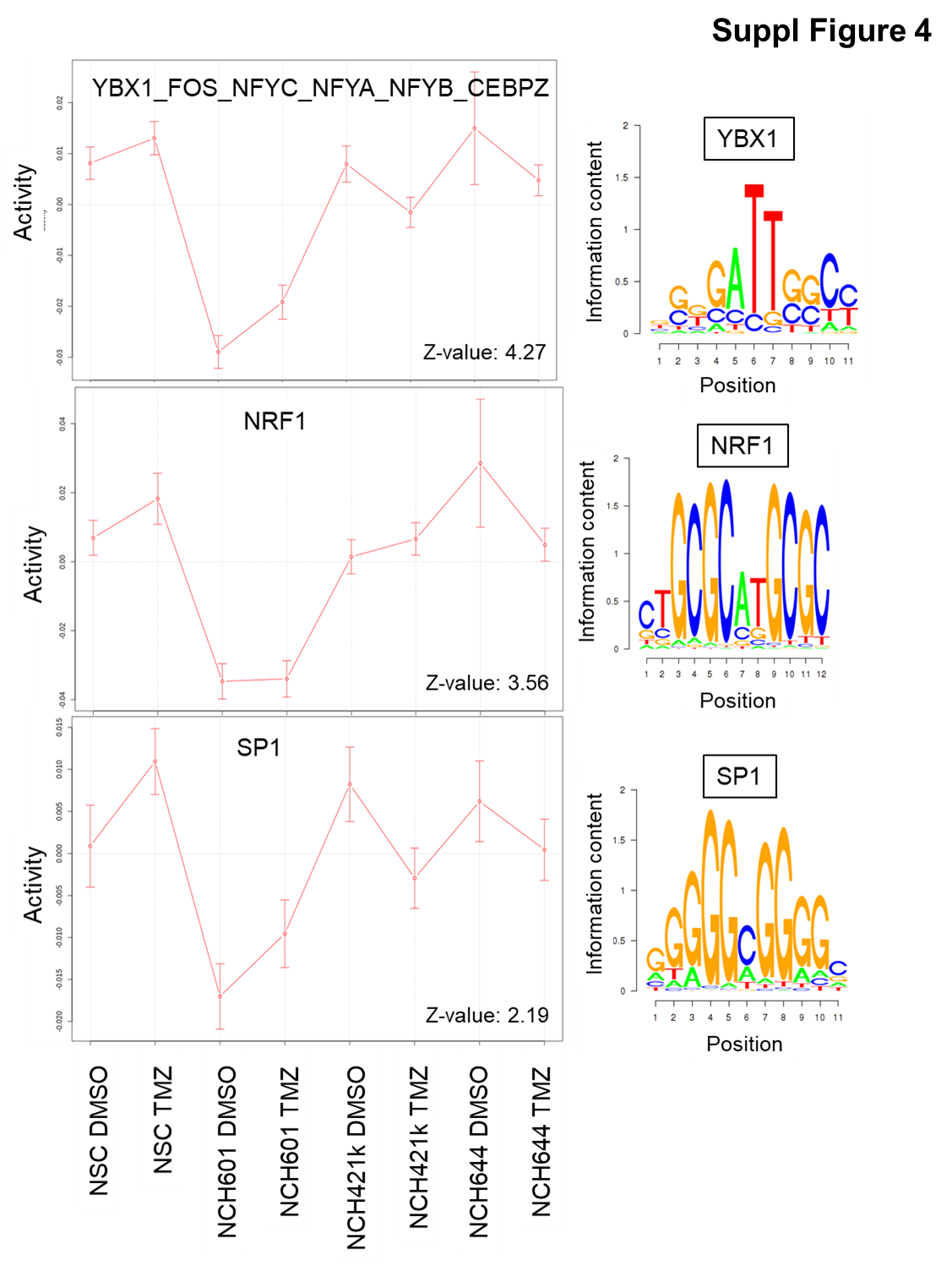


**Figure S4**. Selected examples of Top 10 transcription factor motifs sorted by activities (*z*-value) from ISMARA.


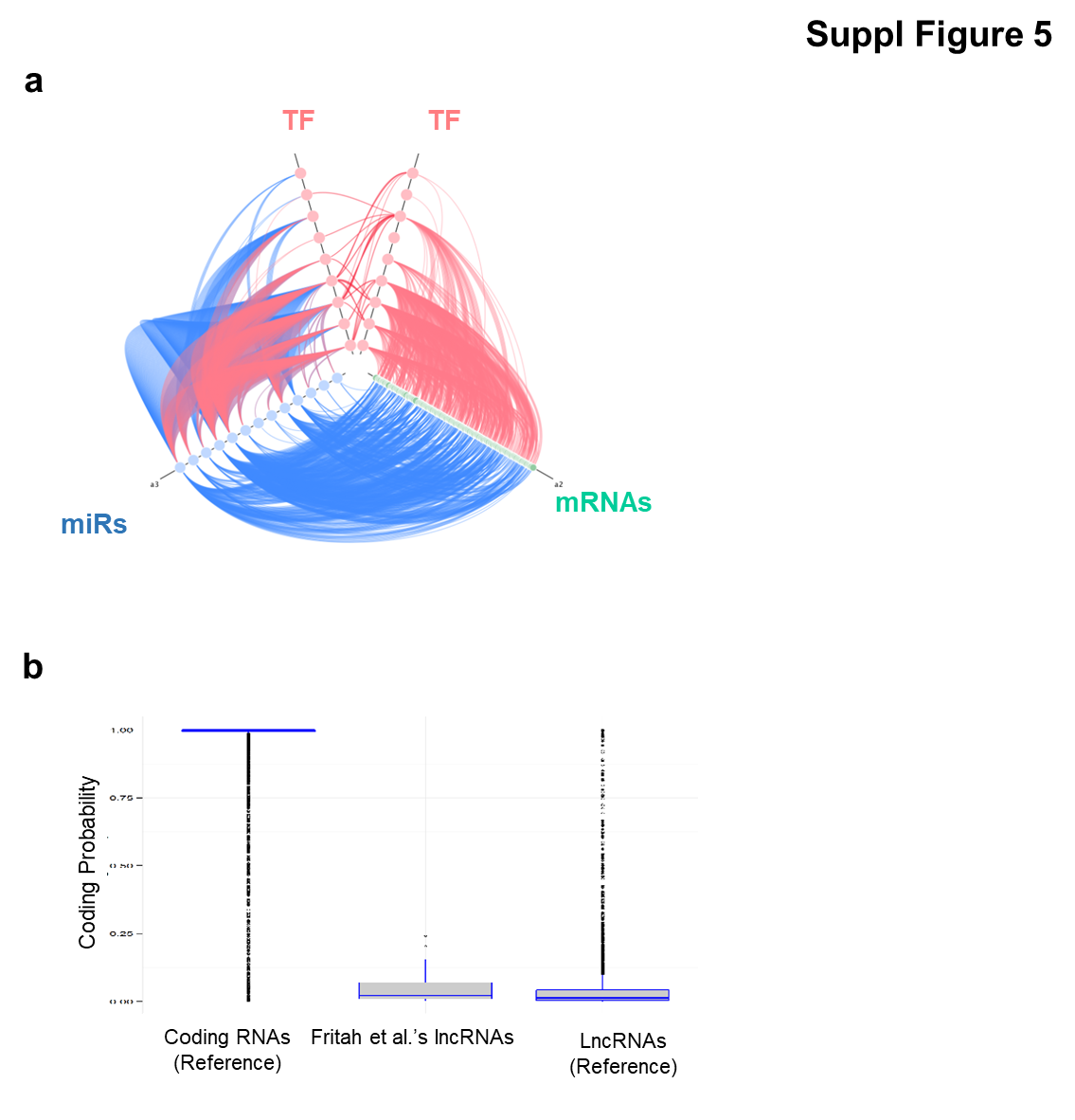


**Figure S5.** mRNA-containing regulatory loops and coding potential of selected lncRNAs (**a**) Hive plot representing TMZ-regulated FFLs containing mRNAs from NCH601. Axes indicate different RNA families, with each dot corresponding to a gene (TF, miRNA, or mRNA) involved in mRNA-containing loops. Molecular interactions are represented by a colour code line (stimulatory interactions in red, inhibitory interactions in blue), (**b**) Distribution of the coding potential of the 22 lncRNAs present in the TMZ- associated motifs predicted by CPAT.


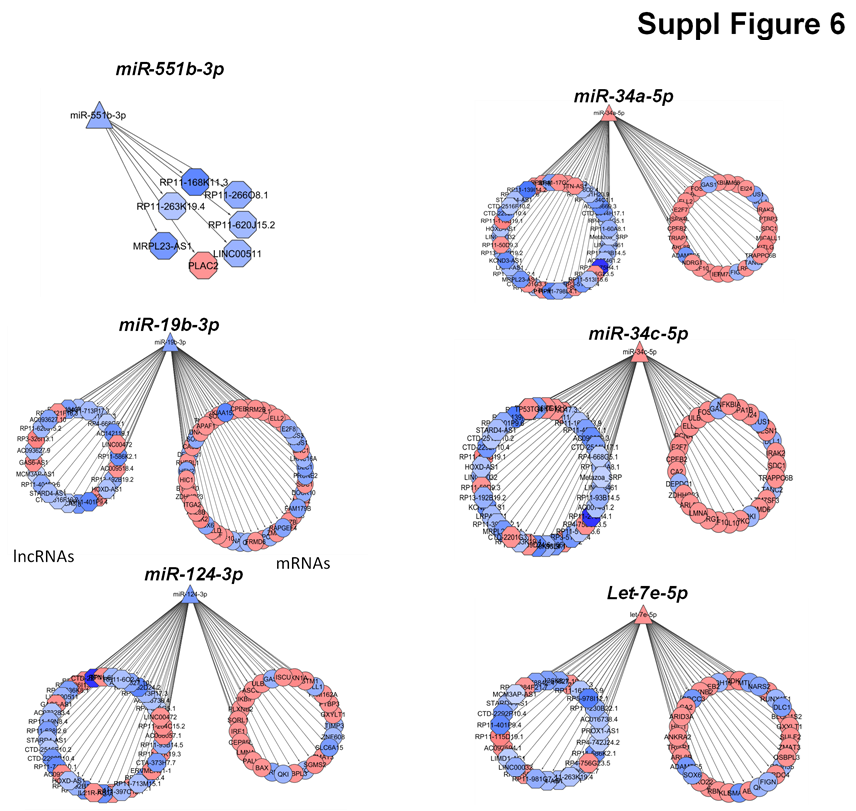


**Figure S6.** Gene regulatory networks representing the molecular associations between selected miRNAs and mRNAs or lncRNAs in NCH601. Up- and downregulated RNAs are shown in red and blue, respectively. Different node shapes distinguish RNA families; TF: square, miRNA: triangle, mRNA: circle, lncRNA: hexagon.
